# Analgesic Activity of Cinnabarinic Acid in Models of Inflammatory and Neuropathic Pain

**DOI:** 10.3389/fnmol.2022.892870

**Published:** 2022-06-02

**Authors:** Serena Notartomaso, Serena Boccella, N. Antenucci, Flavia Ricciardi, Francesco Fazio, F. Liberatore, P. Scarselli, M. Scioli, Giada Mascio, V. Bruno, Giuseppe Battaglia, Ferdinando Nicoletti, Sabatino Maione, Livio Luongo

**Affiliations:** ^1^Department of Molecular Pathology, Istituto di Ricovero e Cura a Carattere Scientifico (IRCCS) Neuromed, Pozzilli, Italy; ^2^Department of Experimental Medicine, University of Campania Luigi Vanvitelli, Naples, Italy; ^3^Department of Physiology and Pharmacology, Sapienza University of Rome, Rome, Italy; ^4^Department of Psychiatry and Health Science, University of California, San Diego, La Jolla, CA, United States; ^5^Istituto di Ricovero e Cura a Carattere Scientifico (IRCCS) Neuromed, Pozzilli, Italy

**Keywords:** analgesia, neuropathic pain, cinnabarinic acid, metabotropic glutamate receptor 4, inflammatory pain

## Abstract

Cinnabarinic acid (CA) is a trace kynurenine metabolite, which activates both type-4 metabotropic glutamate (mGlu4) and arylic hydrocarbon (Ah) receptors. We examined the action of CA in models of inflammatory and neuropathic pain moving from the evidence that mGlu4 receptors are involved in the regulation of pain thresholds. Systemic administration of low doses of CA (0.125 and 0.25 mg/kg, i.p.) reduced nocifensive behaviour in the second phase of the formalin test. CA-induced analgesia was abrogated in mGlu4 receptor knockout mice, but was unaffected by treatment with the Ah receptor antagonist, CH223191 (1 mg/Kg, s.c.). Acute injection of low doses of CA (0.25 mg/kg, i.p.) also caused analgesia in mice subjected to Chronic Constriction Injury (CCI) of the sciatic nerve. Electrophysiological recording showed no effect of CA on spinal cord nociceptive neurons and a trend to a lowering effect on the frequency and duration of excitation of the rostral ventromedial medulla (RVM) ON cells in CCI mice. However, local application of CH223191 or the group-III mGlu receptor antagonist, MSOP disclosed a substantial lowering and enhancing effect of CA on both populations of neurons, respectively. When repeatedly administered to CCI mice, CA retained the analgesic activity only when combined with CH223191. Repeated administration of CA *plus* CH223191 restrained the activity of both spinal nociceptive neurons and RVM ON cells, in full agreement with the analgesic activity. These findings suggest that CA is involved in the regulation of pain transmission, and its overall effect depends on the recruitment of mGlu4 and Ah receptors.

## Introduction

The kynurenine pathway of tryptophan metabolism generates a number of neuroactive compounds, and, therefore, has been extensively studied in experimental animal models of CNS disorders in the last several years. In the first step of the pathway, L-tryptophan is converted by type-1 and -2 indoleamine 2,3-dioxygenase (IDO-1 ad -2) or by tryptophan 2,3-dioxygenase into formyl-kynurenine, which is transformed into L-kynurenine. IDO-1 and -2 are induced by proinflammatory cytokines. L-kynurenine can be transformed into 3-hydroxykynurenine by kynurenine monooxygenase (KMO), or, alternatively, it can be transaminated into kynurenic acid (KYNA) by different isoforms of kynurenine aminotransferase (KAT). 3-Hydroxykyurenine is converted by kynureninase into 3-hydroxyanthranilic acid or, alternatively, is converted by KATs into xanthurenic acid (XA). 3-Hydroxyanthranilic acid can be converted in two steps into quinolinic acid (QUINA), which is further metabolized to produce nicotinic acid ribonucleoside, the precursor of nicotinamide adenine dinucleotide (NAD). Alternatively, 3-hydroxyanthraylic acid may give rise to cinnabarinic acid (CA), a trace kynurenine metabolite, which is increasingly attracting the interest of neurobiologists (Schwarcz et al., 2012). QUINA is an orthosteric agonist of NMDA receptors, whereas KYNA acts as an antagonist at the glycine site of NMDA receptors (Stone, 1993). At least in one study, XA was found to activate type-2 and -3 metabotropic glutamate receptors (mGlu2 and -3 receptors) (Fazio et al., 2017). CA is a pleiotropic molecule, and its action is only partially elucidated. We have found that CA behaves as an orthosteric agonist of mGlu4 receptors (Fazio et al., 2012), a presynaptic receptor that negatively modulates glutamate release (Nicoletti et al., 2011). However, CA can also interact with the aryl hydrocarbon receptor (Ah receptor), a transcription factor known for its ability to bind arylic xenobiotics, such as benzo(a)pirene (Lowe et al., 2014). A large body of evidence supports the involvement of the kynurenine pathway in the regulation of pain thresholds and in the pathophysiology of chronic pain. Both L-kynurenine and 3-hydroxykynurenine are detectable in dorsal horns of the spinal cord (Godefroy et al., 1990), and a number of kynurenine metabolites, including L-kynurenine, KYNA, QUINA, and anthranilic acid modulate pain thresholds in the hot plate ad tail flick tests (Heyliger et al., 1998). In the chronic constriction injury (CCI) model of neuropathic pain, IDO-2, KMO, and 3-hydroxyanthranilate dioxygenase (HAOO) were found to be up-regulated in the spinal cord as result of neuroinflammation and microglia activation, and IDO-2 and KMO inhibitors attenuated mechanical and thermal hyperalgesia (Ciapała et al., 2021). Similar findings were obtained using the KMO inhibitor, Ro 61-8048, in a rat model of neuropathic pain (Laumet et al., 2017). It is generally believed that the influence of the kynurenine pathway on pain transmission is mediated by QUINA or KYNA acting at NMDA receptors and that, for example, KMO inhibitors cause analgesia by shunting L-kynurenine metabolism toward the formation of KYNA (Németh et al., 2005). However, there is increasing evidence that mGlu4 receptors, which are targeted by CA, modulate pain thresholds in different stations of the pain neuraxis, with the overall effect of reducing pain (Pereira and Goudet, 2019). Cyril Goudet, Jean-Philippe Pin, and their associates were first to demonstrate that mGlu4 receptors are located on nerve terminals of unmyelinated C fibres in the dorsal horns of the spinal cord where they negatively modulate glutamate release through coupling to Ca_*v*_2.2 voltage-sensitive calcium channels (Goudet et al., 2009). In addition, they found that genetic deletion of mGlu4 receptors enhances nocifensive behaviour in the formalin test and that pharmacological activation of mGlu4 receptors with subtype-selective agonists attenuates hyperalgesia in animal models of inflammatory or neuropathic pain (Vilar et al., 2013). An elegant follow-up of these findings was the demonstration that mGlu4 receptors are localized on neurons neighbouring intercalated cell clusters in the mouse amygdala, and that optical control of endogenous mGlu4 receptors with a photo switchable positive allosteric modulator rapidly and reversibly inhibited sensory and emotional behaviours associated with persistent inflammatory pain (Zussy et al., 2018). mGlu4 and other group-III mGlu receptors are also found in brainstem regions involved in the top-down control of pain, such as the periaqueductal grey (PAG) and the rostral ventromedial medullary (RVM) nucleus, where they modulate the activity of ON and OFF cells (Ohishi et al., 1995; Drew and Vaughan, 2004). This gave us the impetus to examine the effect of CA in two established mouse pain models: the formalin model of inflammatory pain and the CCI model of neuropathic pain. Using the latter model, we extended the analysis to *in vivo* recording of secondary order sensory neurons in the dorsal horn of the spinal cord and ON and OFF cells in the RVM in an attempt to disclose the electrophysiological mechanisms underlying the effect of CA on pain transmission.

## Materials and Methods

### Drugs

CA and CH223191 were purchased by Sigma-Aldrich (Italy). MSOP was purchased by Tocris Cookson Ltd., (Bristol, United Kingdom). For systemic treatments, CA was dissolved in saline and administered i.p. at doses ranging from 0.125 to 3 mg/Kg (Ulivieri et al., 2020), whereas, CH223191 was dissolved in sesame oil and administered s.c. at the dose of 1 mg/Kg (Ulivieri et al., 2020). For local applications in the VL-PAG or spinal cord, CH223191 and MSOP were dissolved in ACSF containing 0.05% DMSO and used at concentrations of 30 nM and 100 uM, respectively. The concentration of MSOP was chosen on the basis of previous studies in which MSOP was microinjected into the VL-PAG and showed no effect on its own (Maione et al., 1998; Berrino et al., 2001; Palazzo et al., 2001; Marabese et al., 2007a,b). Control mice received the same volume of vehicle (ACSF containing 0.05% DMSO).

### Animals

Experiments were performed following the Guidelines for Animal Care and Use of the National Institutes of Health to minimize the number of animals and animal suffering. The experimental protocol was approved by the Ethical Committee of Neuromed Institute (Pozzilli, Italy), by the Italian Ministry of Health (515/2021-PR), and by the Ethical Committee of University of Campania “Luigi Vanvitelli” (Naples, Italy) and by the Italian Ministry of Health (713/2021-PR). All efforts were made to minimize animal suffering and the number of animals used. In most of the experiments, we used adult male C57BL/6J mice (20–25 g, b.w.) and mGlu4 receptor knockout mice (B6.129-Grm4tm1Hpn/J). Mice were housed in an animal care facility at 23°C on a 12 h light/12 h dark cycle with food and water provided *ad libitum.*

### Drug Treatment

In the formalin test, CA was administered i.p. at doses of 0.125, 0.25, 0.5, or 3 mg/kg always 5 min prior to formalin injection (see below). The respective controls were treated with vehicle. In one experiment, mice were treated s.c. CH223191 (1 mg/kg) or vehicle followed, 5 min later, by i.p. injections of CA (0.25 mg/kg) or vehicle. In another experiment, CA (0.25 mg/kg) or vehicle were injected i.p. in wild-type (WT) and mGlu4^–/–^ mice.

In CCI mice we tested the effects of single or repeated injections of CA (0.25 mg/kg) on mechanical pain thresholds and activity of nociceptive spinal cord neurons and ON RVM cells. In experiments with single injections, either vehicle or CA were administered once i.p. and mechanical thresholds were measured every 15 min from 15 to 120 min following injections. For electrophysiological analysis, either MSOP or CH223191 were locally applied in the spinal cord or in the VL-PAG. In experiments with repeated injections, vehicle, s.c. + vehicle, i.p.; vehicle, s.c. + CA (0.25 mg/kg), i.p.; CH223191 (1 mg/kg), s.c., + vehicle, i.p.; or CH223191, s.c. + CA, i.p. were administered once a day for 7 days starting from day 7 following nerve ligation. Subcutaneous injections were always carried out 5 min prior to i.p. injections. Mechanical thresholds were determined only once 15 min after the last i.p. injection.

### Formalin Test

Acute inflammatory pain was assessed using the formalin test. Ten μl of a formalin solution (2%) was injected s.c. into the plantar surface of the right hind paw. After injection, mice were immediately placed in a plexiglass box (20 cm × 15 cm × 15 cm) surrounded by mirrors to allow the observation of nociceptive responses that include licking, lifting and shaking of the injected paw. After formalin injection, mice were observed for 1 h and their behaviour was recorded by researchers blind to genotypes and drug treatments. Formalin scores were separated into two phases, phase I (0–10 min) and phase II (30–60 min).

### Induction of Chronic Constriction Injury of the Sciatic Nerve

Chronic constriction of the sciatic nerve was carried out under isoflurane anesthesia (5% for induction and 2% for maintenance), as described by Bennett and Xie (1988). In brief, the biceps femoris and the gluteus superficialis were separated by blunt dissection, and the left sciatic nerve was exposed. CCI was produced by tying two ligatures around the sciatic nerve. The ligatures were tied loosely around the nerve with 1 mm spacing, until they elicited a brief twitch in the respective hind limb, which prevented over-tightening of the ligations, taking care to preserve epineural circulation. The incision was cleaned and the skin was closed with 2–3 ligatures of non-absorbable silk suture size 5–0. Mice were then placed on a warmed surface and, following recovery, they were returned to their home cages and checked routinely for 72 h. In Sham-operated mice (SO) mice, the left sciatic nerve was exposed without ligature. Mechanical allodynia (see below) was assessed 14 days after surgery. The development of mechanical allodynia was evaluated by using the von Frey filaments. All animals were tested before surgery and then 14 days after surgery. Mechanical allodynia was quantified by measuring the hind paw withdrawal response to von Frey filament stimulation.

### Assessment of Mechanical Allodynia (Von Frey Test)

Mechanical allodynia was measured by a series of calibrated von Frey filaments (Stoelting, Wood Dale, IL, United States), ranging from 0.02 to 2 gr. Neuropathic mice were placed in plastic cages with a wire-mesh floor approximately 1 h before testing to allow behavioural accommodation. The von Frey filaments were applied in ascending order to the mid-plantar surface of the hind paw through the mesh floor. If the use of the filament three times did not induce a reaction, the next filament with higher pressure was used. The time interval before the application of each filament was at least 5 s. Data were expressed as mean ± S.E.M. of the mechanical withdrawal threshold (MWT) in grams.

### Extracellular Recordings of Spinal Nociceptive Specific Neurons *in vivo*

For *in vivo* single unit extracellular recording, mice were initially anesthetized with Avertin (1.25%). After tracheal cannulation, a catheter was placed into the right external jugular vein to allow continuous infusion of propofol (5–10 mg/kg/h, i.v.). Spinal cord segments L4–L6 were exposed medially by laminectomy, near the dorsal root entry zone, up to a depth of 1 mm. An elliptical rubber ring (about 3 mm × 5 mm) was tightly sealed with silicone gel onto the surface of the cord. This ring formed a trough with about 50 μL capacity over the spinal segments used for topical spinal drug application.

It also provided access to the spinal neurons that receive input from the ipsilateral paw, where the mechanical stimulation was applied. Animals were then secured in a stereotaxic apparatus (David Kopf Instruments, Tujunga, CA, United States) supported by clamps attached to the vertebral processes on either side of the exposure site. The exposed area of the spinal cord was initially framed by agar and then filled with mineral oil. Body temperature was maintained at 37°C with a temperature-controlled heating pad. A glass-insulated tungsten filament electrode (3–5 MΩ; FHC Frederick Haer & Co., Bowdoin, ME, United States) was used to record single unit extracellular activity of dorsal horn NS neurons. NS neurons were defined as those neurons that respond only to high-intensity (noxious) stimulation. To confirm NS response patterns, each neuron was characterized while applying a mechanical stimulation to the ipsilateral hind paw using a von Frey filament with 97.8 mN bending force (noxious stimulation) for 2 s until it buckled slightly. Only neurons that responded specifically to the noxious hind paw stimulation, without responding to stimulation of the surrounding tissue, were included in sham and neuropathic mice recordings. The recorded signals were amplified and displayed on a digital storage oscilloscope to ensure that the unit under study was unambiguously discriminated throughout the experiment. Signals were also fed into a window discriminator, whose output was processed by an interface CED 1401 (Cambridge Electronic Design Ltd., Milton, United Kingdom) connected to a Pentium III PC. Spike2 software (CED, version 4) was used to create peristimulus rate histograms online and to store and analyse digital records of single unit activity offline. Configuration, shape, and height of the recorded action potentials were monitored and recorded continuously using a window discriminator and Spike2 software for online and offline analysis. This study only included neurons whose spike configuration remained constant and could be clearly discriminated from activity in the background throughout the experiment, indicating that the activity from one neuron only and from the same neuron was measured. The neuronal activity was expressed as spikes/s (Hz). At the end of the experiment, each animal was killed with a lethal dose of urethane. The spontaneous and noxious-evoked neuronal activity was expressed as spikes/s (Hz) and the effect of drugs was analyzed as % variation of firing rate, frequency and duration of excitation. For acute experiments, after recording a stable basal activity (10 min), topical spinal application of vehicle or drugs was performed, and each extracellular recording was monitored until 45-60 min post-injection. In particular, groups of animals were divided as following: (1) Vehicle (sesame oil or 0.05% DMSO in ACSF), (2) Cinnabarinic acid (CA) 0.25 mg/Kg (the anti-nociceptive or analgesic dose), (3) MSOP (100 uM), (4) MSOP (100 uM/5 ul)+CA (0.25 mg/Kg, i.p.), (5) CH223191 (30 nM) and (6) CH223191 (30 nM)+ CA 0.25 mg/Kg (i.p.). Vehicle or drugs were applied on the spinal cord in a volume of 5 μl and at the end of the experiment, each animal was killed with a lethal dose of urethane.

### Surgical Preparation for Intra-PAG Microinjections

Control and CCI mice were anesthetized with pentobarbital (50 mg/kg, i.p.), and a 26-gauge, 8 mm-long stainless steel guide cannula was stereotaxically lowered until its tip was 1.0 mm above the VL PAG (AP: -4.7 mm and L: 0.5 mm from bregma, V: 1.78 mm below the dura) (Paxinos and Franklin, 2001). The cannula was anchored with dental cement to a stainless steel screw in the skull. We used a David Kopf stereotaxic apparatus (David Kopf Instruments, Tujunga, CA, United States) with the animal positioned on a homeothermic temperature control blanket (Harvard Apparatus Limited, Edenbridge, United Kingdom). Intra-VL PAG administration was conducted with a stainless steel cannula connected by a polyethylene tube to an SGE 1-μl syringe, inserted through the guide cannula, and extended 1.0 mm beyond the tip of the guide cannula to reach the VL PAG. Groups of animals were divided as following: (1) Vehicle (sesame oil or 0.05% DMSO in ACSF), (2) CA 0.25 mg/Kg (the anti-nociceptive or analgesic dose), (3) MSOP (100 uM), (4) MSOP (100 uM/5ul)+CA (0.25 mg/Kg, i.p.), (5) CH223191 (30 nM), and (6) CH223191 (30 nM)+ CA 0.25 mg/Kg (i.p.). Vehicle or drugs were injected in a volume of 0.5 μl into the VL PAG for 60 s and the injection cannula gently removed 2 min later. At the end of the experiment, each animal was killed with a lethal dose of urethane.

### Extracellular Recordings of Rostral Ventromedial Medulla ON Cell *in vivo*

Single unit extracellular recordings have been carried out in the RVM while microinjecting MSOP, CH223191 or vehicle into the VL PAG in sham and CCI mice. We implanted the cannula in the ventrolateral subregion of the PAG because output neurons from VL PAG project directly to RVM (Sandkühler and Gebhart, 1984; Moreau and Fields, 1986). A glass-insulated tungsten filament electrode (3–5 MW) (FHC Frederick Haer& Co., Bowdoin, ME, United States) was lowered into the RVM using the stereotaxic coordinates (AP: -6.48 mm and L: 0.3–0.5 mm from bregma, and V: 4.5–6.0 mm below the dura) from the atlas of Paxinos and Franklin (2001). Pain-responding neurons were identified by the characteristic ON cell burst of activity evoked by the noxious stimulus. In particular, neurons showing an abrupt acceleration of firing activity just prior to the pain-evoked nocifensive reaction were identified as ON cells. Neurons showing no change in activity just before the nocifensive reaction were identified as neutral cells and were not recorded (Fields et al., 1983). The recorded signals were amplified and displayed on both, analogue and a digital storage oscilloscope to ensure that the unit under study was unambiguously discriminated throughout the experiment. Signals were sampled by a CED 1401 interface (Cambridge Electronic Design Ltd., United Kingdom) and analyzed by Spike2 window software (CED, version 4) to create peristimulus rate histograms online and to store and analyze digital records of single-unit activity offline. The configuration, shape, and height of the recorded action potentials were monitored and recorded continuously using Spike2 software for online and offline analyses. Once an ON cell was identified from its background activity, we optimized spike size before all treatments. This study only included neurons whose spike configuration remained constant and could clearly be discriminated from the background activity throughout the entire experiment. By doing so, we were able to determine the activity of a single neuron only. In each mouse, the activity of only a single neuron was recorded before and after vehicle or drug administration. The ongoing activity, the average of the firing rate (spikes/s) for 50 s before the noxious stimulus (which was carried out every 5 min), and noxious stimulus-evoked activity, the peak height (spikes/s) of the noxious stimulus-evoked burst and the onset of the ON cell burst (the time elapsing between the onset of noxious stimulus application and the increase in the frequency rate, which was at least twofold higher than its baseline) were quantified for the ON cells, before and after the VL PAG microinjection of vehicle or drugs. At the end of the experiment, a volume of 200 nl of neutral red (0.1%) was injected into the VL PAG for 30 min before killing the mice with a lethal dose of urethane. Mice were then perfused intracardially with 20 ml phosphate buffer solution (PBS) followed by 20 ml of 10% formalin solution in PBS. The brains were removed and immersed in a saturated formalin solution for 2 days. After fixation, the microinjection and recording sites were identified. The injection sites were ascertained using two consecutive sections (40 μm), one stained with cresyl violet to identify the microinjection site within the VL PAG, and the other unstained to determine dye spreading. The recording site was marked with a 20 μA DC current applied for 20 s immediately prior to the end of the electrophysiological recordings. Only the data from drug microinjection and diffusion sites located within the VL PAG and those from the recording sites in RVM neurons were included in the results.

### Statistics

Statistical analysis was performed using GraphPad Prism 9 (GraphPad Software). Behavioural data were expressed as mean ± standard error of the mean (SEM). Electrophysiological data were represented as percentage change from baseline. Test for normal distribution was performed by Kolmogorov-Smirnov test with Lilliefors significance correction. The *t-*test was used to test differences between two means of paired/unpaired data. Under condition of normal distribution, ANOVA for repeated measures was performed followed by Sidak’s *post-hoc* test to compare post-injection *vs.* pre-injection values or Tukey’s *post-hoc* test to calculate significant differences between the different groups of mice. One Two-way ANOVA followed by Tukey’s *post-hoc* test for multiple comparisons was used. *P* < 0.05 was considered statistically significant. Using G*Power3 software (Faul et al., 2007) with α = 0.05 and power equal to 80%, sample size requirements for testing the efficacy of CA at 0.25 mg/Kg was *n* = 5.

## Results

### Effect of Cinnabarinic Acid in the Formalin Test

#### Inflammatory Pain in C57BL/6J Mice, Wild-Type, and mGlu4^–/–^ Mice

**FIGURE 1:**
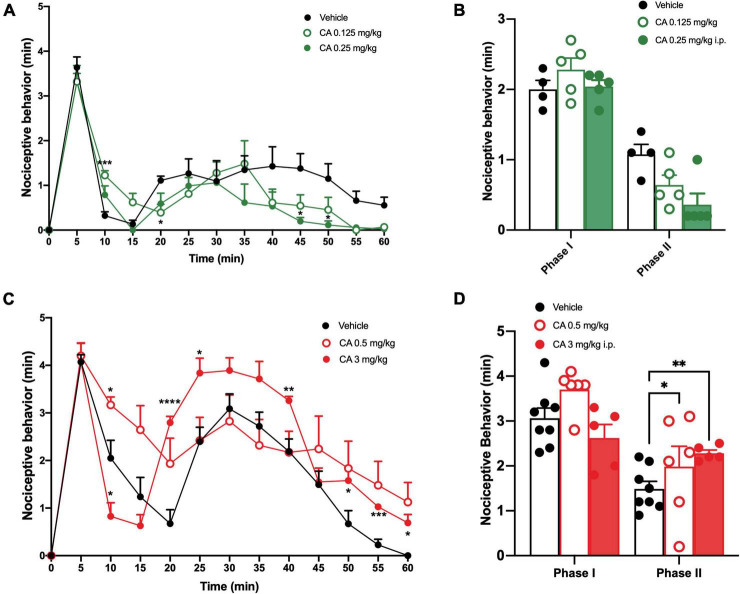
Low doses of cinnabarinic acid significantly reduced formalin-induced nocifensive response in wild-type (WT) mice. **(A)** Effect of lowest doses of cinnabarinic acid (0.125 and 0.25 mg/Kg) on formalin-induced pain behaviours, including lifting, flinching, and licking, observed for 60 min at 5 min intervals. **(B)** Total pain scores were classified as phase I (0–10 min) and phase II (30–60 min) in formalin-injected mice treated with vehicle or with cinnabarinic acid (0.125 and 0.25 mg/Kg). **(C)** Effect of highest doses of cinnabarinic acid (0.5 and 3 mg/Kg) on formalin-induced pain behaviours, including lifting, flinching, and licking, observed for 60 min at 5 min intervals. **(D)** Total pain scores were classified as phase I (0–10 min) and phase II (30–60 min) in formalin-injected mice treated with vehicle or with cinnabarinic acid (0.5 and 3 mg/Kg). Data are expressed as the means ± SEMs (two-way ANOVA for repeated measures with Tukey’s *post-hoc* test, *p < 0.05, **p < 0.01, and ****p < 0.0001 vs. vehicle, *n* = 6–8).

We induced inflammatory pain by injecting a 2% formalin solution in the right hind paw. Control mice (C57BL/6J mice injected i.p. with vehicle) showed a first phase of nocifensive behaviour in the first 0–10 min after formalin injection, followed by a second phase (30–60 min), which reflects the development of nociceptive sensitization in the dorsal horns of the spinal cord (Coderre and Melzack, 1992; Tjølsen et al., 1992). Very low doses of CA (0.125 or 0.25 mg/kg, i.p.) significantly reduced nocifensive behaviour in the second phase of the formalin test (Figures 1A,B), whereas, doses of 0.5 or 3 mg/kg, i.p. were inactive (Figures 1C,D). Indeed, mice treated with 0.125 or 0.25 mg/kg of CA, showed a nocifensive response in the second phase post-formalin injection of 0.64 ± 0.14 min (*p* = 0.046) and 0.36 ± 0.16 min (*p* = 0.002), respectively, as compared to vehicle-injected mice (1.07 ± 0.14 min) (Figures 1A,B). On the contrary, mice treated with 0.5 or 3 mg/kg of CA, showed a nocifensive response in the second phase post-formalin injection of 1.98 ± 0.45 and 2.28 ± 0.073 min, respectively, as compared to vehicle-injected mice (1.48 ± 0.16 min) (Figures 1C,D).

**FIGURE 2:**
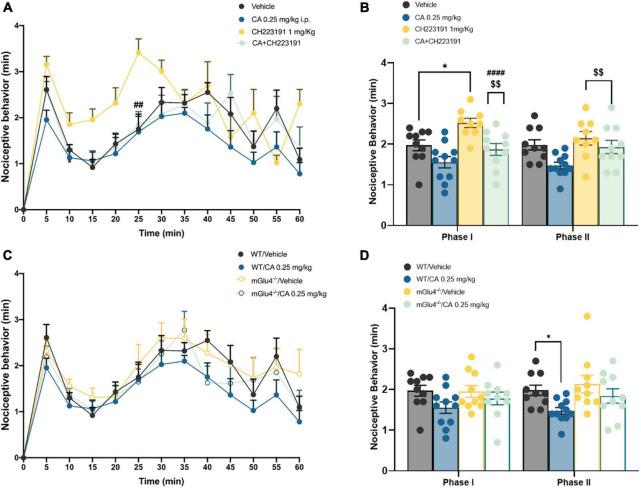
Cinnabarinic acid-induced analgesia was mediated by the activation of mGlu4 receptor in WT and mGlu4^–/–^ mice. **(A)** Effect of cinnabarinic acid (0.25 mg/Kg) alone or in combination with CH223191 (1 mg/Kg) on formalin-induced pain behaviours, including lifting, flinching, and licking, observed for 60 min at 5 min intervals. **(B)** Total pain scores were classified as phase I (0–10 min) and phase II (30–60 min) in formalin-injected mice treated with vehicle or with cinnabarinic acid (0.25 mg/Kg) alone or in combination with CH223191 (1 mg/Kg). **(C)** Effect of cinnabarinic acid (0.25 mg/Kg) on formalin-induced pain behaviours, in WT and in mGlu4^–/–^ mice, observed for 60 min at 5 min intervals. **(D)** Total pain scores were classified as phase I (0–10 min) and phase II (30–60 min) in WT and in mGlu4^–/–^ mice formalin-injected mice treated with vehicle or with cinnabarinic acid (0.25 mg/Kg). Data are expressed as the means ± SEMs (two-way ANOVA for repeated measures with Tukey’s *post-hoc* test, *p < 0.05 vs. vehicle, ^$$^*p* < 0.01 vs. CH223191, and ^####^*p* < 0.0001 vs. CA, *n* = 8–10).

Formalin tests show different nocifensive responses during the first phase, between vehicle or CA-injected mice; this is likely due to diverse experimental settings among research laboratories where studies have been carried out. However, these discrepancies were not statistically different, as reported by t-test analysis. To examine whether CA-induced analgesia was mediated by the activation of mGlu4 receptors, we used mGlu4^–/–^ mice. mGlu4^–/–^ mice treated with vehicle showed no difference in nocifensive behaviour (1.97 ± 0.13 min in the I phase and 1.98 ± 0.12 min in the II phase) with respect to corresponding group of WT mice [see Vilar et al. (2013); Figures 2A,B]. Systemic injection of low doses of CA (0.25 mg/kg, i.p.) reduced nocifensive behaviour in WT (1.47 ± 0.08 min, *p* = 0.047), but not in mGlu4^–/–^ mice (1.84 ± 0.17 min, *p* = 0.56) (Figures 2A–D). These findings suggest that, at least in the formalin test, CA-induced analgesia was mediated by the activation of mGlu4 receptors.

We also examined whether Ah receptor activation could contribute to the overall effect of CA in the formalin test by injecting C57BL/6J mice with the Ah receptor antagonist, CH-223191 (1 mg/kg, s.c.). This treatment caused a slight, but significant increase in nocifensive behaviour in the second phase of the formalin test (1.93 ± 0.16 min, *p* = 0.048) (Figures 2C,D). Low doses of CA (0.25 mg/kg) caused a similar reduction in nocifensive behaviour in control mice and in mice pre-treated with CH-223191 (2.14 ± 0.16 min, *p* = 0.68), indicating that the Ah receptor was not involved in CA-induced analgesia in the formalin test (Figures 2A,B).

Taken together these findings demonstrate that: (i) CA causes analgesia in the second phase of the formalin test; (ii) that CA is active at very low doses; and, (iii) that the action of CA in this test requires the presence of mGlu4 receptors and does not involve the Ah receptor.

### Effect of Acute Cinnabarinic Acid Injection in the Chronic Constriction Injury Model of Neuropathic Pain

#### Mechanical Pain Thresholds in Wild-Type Chronic Constriction Injury Mice

**FIGURE 3:**
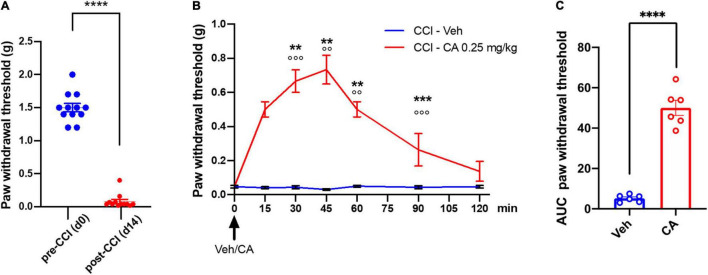
Single injection of cinnabarinic acid showed analgesic effect in WT CCI mice. **(A)** Paw withdrawal thresholds in CCI mice recorded before (day 0) and after (day 14) CCI surgery. Data are expressed as the means ± SEMs [paired *t*-student test, ****p < 0.0001 vs. pre-CCI (d0), *n* = 12]. **(B)** Time-course of paw withdrawal threshold (g) measurements before and after single injection of vehicle or CA (0. 25 mg/Kg) in CCI mice, recorded for 120 min at 15 min intervals. Data are expressed as the means ± SEMs [two-way ANOVA for repeated measures with Tukey’s or Sidak *post-hoc* test for differences within and between group of animals, **p < 0.01, ***p < 0.001 and ****p < 0.0001 vs. pre-drug (time 0), °°*p* < 0.01 and °°°*p* < 0.001 vs. vehicle, *n* = 6]. **(C)** The area under curve (AUC) analysis (normalized to -1 d values) of paw withdrawal threshold differences in between vehicle and CA-injected CCI mice. Data are expressed as the means ± SEMs (unpaired *t*-student test, ****p < 0.0001 vs. CCI-Veh, *n* = 6).

Mice were subjected to CCI and treated i.p. with an acute injection of either saline or CA (0.25 mg/kg) after 14 days, when mechanical pain thresholds were markedly reduced as compared to basal values (0.11 ± 0.06 g *vs.* 1.5 ± 0.07 g, *p* < 0.0001) (Figure 3A). Treatment with CA, 14 days-post-CCI, caused substantial analgesia from 15 min up to 60 min post-CA, as compared to vehicle-treated mice (0.73 ± 0.084 g *vs.* 0.030 ± 0.084 g, *p* = 0.0032, at 45 min post-injection corresponding to peak of analgesia induced by CA) and it returned back to baseline values at 120 min (Figure 3B). Calculation of the AUC, as a measure of the effect of CA during the complete period of observation, showed that the antiallodynic effect of cinnabarinic acid resulted statistically significant (*p* < 0.0001) (Figure 3C).

#### Electrophysiological Activity of Spinal Nociceptive Specific Neurons in Wild-Type Chronic Constriction Injury Mice

In addition, to examine the regional specificity of the analgesic action of acute CA injection we recorded neuronal activity in two regions of the pain neuraxis: the dorsal horns of the spinal cord, and the RVM.

**FIGURE 4:**
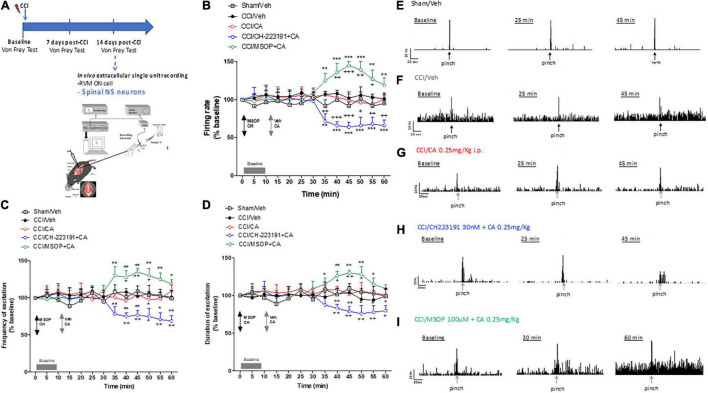
Spinal cord neuronal hyperexcitability is oppositely modulated by mGlu4 or aryl hydrocarbon receptor in WT CCI mice. **(A)** Experimental timeline (top) and graphical representation of extracellular recordings of spinal NS neurons in anesthetized mice. **(B–D)** Mean ± S.E.M population data of spinal cord application of MSOP (100 uM) or CH223191 (30 nM) in presence of single systemic injection of cinnabarinic acid (CA, 0.25 mg/Kg) on firing rate **(B)**, frequency **(C)**, and duration of excitation **(D)** of NS neurons, in CCI mice. Black arrows indicate MSOP or CH223191 spinal application. Grey arrows indicate vehicle or CA spinal application. Grey bars indicate interval recordings of basal neuronal activity. Each point represents the mean of five different mice per group (one neuron recorded per each mouse). *p < 0.05, **p < 0.01, and ***p < 0.001 indicate statistically significant difference vs. baseline, ^+^*p* < 0.05, ^++^*p* < 0.01, and ^+++^*p* < 0.001 indicate statistically significant difference vs. CCI/CA. Two-way ANOVA for repeated measures followed by Tukey’s *post-hoc* test was used for comparison between groups. Two-way ANOVA for repeated measures followed by Sidak *post-hoc* test was used for comparison between pre-drug and post-drug values. **(E–I)** Representative ratemeters showing spontaneous and noxious-evoked activity of NS neurons in Sham mice **(E)** or in CCI mice before and after treatment with vehicle **(F)**, CA (0.25 mg/Kg) **(G)**, CH223191+CA **(H)**, and MSOP+CA **(I)**.

We recorded the activity of NS neurons (one cell recorded from each animal per treatment) at a depth of 0.7–1.0 mm from the surface of the spinal cord. This cell population was characterized by a mean rate of spontaneous firing of 0.015 ± 0.002 spikes/s and only cells showing this pattern of basal firing were chosen for the experiment. We confirmed an overall NS neuron hyperexcitability in CCI mice as compared with sham-operated mice (mice which underwent surgery but not nerve ligation) [see Boccella et al. (2015)]. In particular, we found a significant increase in spontaneous activity (12.33 ± 2.7 spikes/sec, *p* = 0.003, *n* = 5), and in the frequency (29 ± 3.2 spikes/sec, *p* = 0.006, *n* = 5) and duration of the excitation (11 ± 2.2 sec, *p* = 0.003, *n* = 5) of NS neurons, 14 days after CCI (Figures 4B–D,F). Sham-operated animals showed a firing rate of 0.1 ± 0.03 spikes/s (*p* = 0.003, *n* = 5), a frequency of excitation of 10.7 ± 1.2 spikes/s (*p* = 0.003, *n* = 5), and a duration of excitation of 3.6 ± 0.9 s (*p* = 0.003, *n* = 5) (Figures 4B–E). A single injection of CA (0.25 mg/kg, i.p.) did not change any of these parameters in CCI mice. In particular, NS neurons showed a mean percentage of spontaneous activity of 102.9 ± 5% (*p* = 0.99), a frequency of excitation of 96.19 ± 8% (*p* = 0.85) and a duration of excitation of 108.12 ± 4% (*p* = 0.95) 30 min following CA injection (Figures 4B–D), a time at which CA-induced analgesia was substantial (Figures 4B–D,G). We therefore examined whether pharmacological manipulation of spinal mGlu4 or Ah receptor receptors could disclose an activity of systemic CA on spinal NS neurons of CCI mice. We topically applied either the group-III mGlu receptor antagonist, MSOP (100 μM), or the Ah receptor antagonist, CH223191 (30 nM) 10 min prior to systemic CA administration. Neither MSOP nor CH223191 showed any effect on the activity of NS neurons prior to CA injection. However, spinal application of MSOP significantly increased the mean percentage of firing rate (136 ± 12%, *p* = 0.0002, *n* = 5), frequency of excitation (128 ± 9%, *p* = 0.006, *n* = 5) and the duration of excitation (126 ± 6%, *p* = 0.0009, *n* = 5) in CCI mice starting from 20 min post-CA injection until the end of recording (60 min), as compared to CCI mice injected with CA alone (Figures 4B–D,I). Opposite results were obtained with CH223191, which significantly reduced the mean frequency of firing rate (66 ± 5%, *p* = 0.0004, *n* = 5), frequency of excitation (75 ± 5%, *p* = 0.0023, *n* = 5) and the duration of excitation (83 ± 4.5%, *p* = 0.004, *n* = 5) in CCI mice starting from 20 min post-CA injection (Figures 4B–D,H).

#### Electrophysiological Activity of the Rostral Ventromedial Medulla ON Cell in Wild-Type Chronic Constriction Injury Mice

**FIGURE 5:**
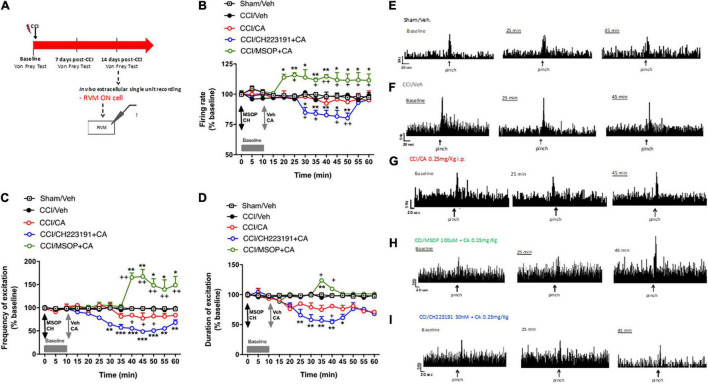
Rostral ventromedial medullary (RVM) ON cell increased activity is oppositely modulated by mGlu4 or aryl hydrocarbon receptor in WT CCI mice. **(A)** Experimental timeline (top) and graphical representation of extracellular recordings of RVM ON cell in anesthetized mice. **(B–D)** Mean ± S.E.M population data of intra vl-PAG of MSOP (100 uM) or CH223191 (30 nM) in presence of single systemic injection of cinnabarinic acid (CA, 0.25 mg/Kg) on firing rate **(B)**, frequency **(C)**, and duration of excitation **(D)** of ON cell, in CCI mice. Black arrows indicate MSOP or CH223191 intra vl-PAG microinjections. Grey arrows indicate vehicle or CA intra vl-PAG microinjections. Grey bars indicate interval recordings of basal neuronal activity. Each point represents the mean of 5 different mice per group (one neuron recorded per each mouse). *textitp < 0.05, **p < 0.01, and ***p < 0.001 indicate statistically significant difference vs. baseline, ^+^*p* < 0.05 and ^++^*p* < 0.01 indicate statistically significant difference vs. CCI/CA. Two-way ANOVA for repeated measures followed by Tukey’s *post-hoc* test was used for comparison between groups. Two-way ANOVA for repeated measures followed by Sidak *post-hoc* test was used for comparison between pre-drug and post-drug values. **(E–I)** Representative ratemeters showing spontaneous and noxious-evoked activity of ON cell in Sham mice **(E)** or in CCI mice before and after treatment with vehicle **(F)**, CA (0.25 mg/Kg) **(G)**, CH223191+CA **(H)**, and MSOP+CA **(I)**.

In a separate experiment, we recorded the activity of ON cells in the RVM after acute systemic injection of CA in CCI mice. In sham-operated mice, the population of ON cells had a firing rate of 7.1 ± 0.4 spikes/s, a burst of excitation of 13.28 ± 0.64 spikes/s and a duration of excitation of 4 ± 0.37 s (*n* = 5) (Figures 5A–C). In CCI mice, the population of ON cells had a firing rate of 16 ± 2 spikes/s (*p* = 0.03, *n* = 5), a frequency of excitation of 26.2 ± 1.4 spikes/s (*p* = 0.005, *n* = 5) and a duration of excitation of 12 ± 2.4 s (*p* = 0.0021, *n* = 5) that were higher compared to sham mice (Figures 5B–D). Systemic injection of CA in CCI mice did not change the firing rate of ON cells (Figure 5B), but caused a trend to a decreased in the frequency (Figure 5C), and duration (Figure 5D) of excitation of ON cells. ON cell showed a mean frequency of spontaneous activity of 92.4 ± 3.59% (*p* = 0.0021, *n* = 5), a frequency of excitation of 83.99 ± 1.74% (*p* = 0.11, *n* = 5) and the duration of excitation of 80.93 ± 0.096% (*p* = 0.095, *n* = 5) of noxious-evoked activity of NS neurons. Recordings of RVM ON cell in CCI mice in which MSOP or CH223191 had been locally applied in the VL-PAG produced results similar to those observed after application of the two drugs in the spinal cord. In particular, intra-PAG microinjection of MSOP (100 uM), 10 min before CA, significantly increased the firing rate (114.67 ± 1.57%, *p* = 0.003, *n* = 5) and the frequency of excitation (165.45 ± 9.54%, p = 0.0022, n = 5), as compared to CCI mice injected with CA alone (Figures 5A–C), whereas only a slight and transient effect was observed on the duration of excitation (109.69 ± 0.1%, *p* = 0.049, *n* = 5) of ON cell in CCI mice (Figures 5B–D). In contrast, intra-PAG microinjection of CH223191 (30 nM), 10 min before CA, caused a significant reduction of all three electrophysiological parameters of RVM ON cell in CCI mice (Figures 5B–D). In particular, ON cell showed a spontaneous activity of 82.69 ± 4% (*p* = 0.038, *n* = 5), a frequency of excitation of 55.57 ± 3.25% (*p* = 0.044, *n* = 5) and the duration of excitation of 54.43 ± 3.58% (*p* = 0.041, *n* = 5) in CCI mice starting from 25 to 30 min post-CA injection until the end of recording (60 min) as compared to CCI mice injected with CA alone (Figures 5A–C). Neither MSOP nor CH223191 caused any change in the activity of RVM ON cells by themselves (i.e., prior to CA administration) (Figures 5B–D). Spontaneous and noxious-evoked activity of a single ON cell in Sham mice (Figure 5E) or in CCI mice before and after treatment with vehicle (Figure 5F), CA (0.25 mg/Kg) (Figure 5G), CH223191+CA (Figure 5H), and MSOP+CA (Figure 5I) are represented by ratematers recordings.

Taken together these findings demonstrate that (i) acute CA administration causes analgesia in the CCI model of neuropathic pain; and, (ii) the effect of acute CA on the activity of nociceptive spinal neurons and RVM ON cells is disclosed by pharmacological blockade of group-III mGlu receptors or Ah receptors.

### Effect of Repeated Administrations of Cinnabarinic Acid in the Chronic Constriction Injury Model of Neuropathic Pain

#### Mechanical Pain Thresholds in Wild-Type Chronic Constriction Injury Mice

**FIGURE 6:**
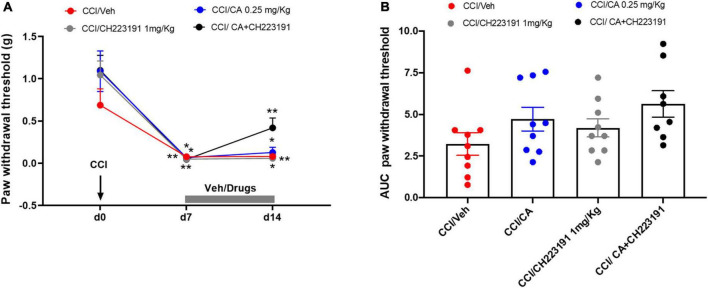
Analgesic activity of chronic treatment with CA was disclosed when the drug was combined with systemic CH223191 in WT CCI mice. **(A)** Effect of repeated administrations of cinnabarinic acid (0.25 mg/Kg) alone or in combination with CH223191 (1 mg/Kg) on paw withdrawal threshold (g) in CCI mice (*n* = 7–9 mice per group). Black arrow indicates chronic constriction surgery (d0). Grey bar indicate the duration of drug treatment (from d7 to d14). **(B)** The area under curve (AUC) analysis (normalized to -1 d values) of paw withdrawal thresholds in CCI mice treated with vehicle, CA, CH223191 or CA+CH223291 (*n* = 7–9 mice per group). Two-way ANOVA for repeated measures followed by Sidak *post-hoc* test was used for comparison between pre-drug and post-drug values. *p < 0.05 and **p < 0.01 indicate statistically significant difference vs. baseline (d0).

Chronic constriction injury mice were treated daily for 7 days with (i) saline, i.p. + sesame oil, s.c.; (ii) CA (0.25 mg/kg), i.p. + sesame oil, s.c.; (iii) saline, i.p. + CH223191 (1 mg/kg), s.c.; or CA (0.25 mg/kg), i.p. + CH223191 (1 mg/kg), s.c., starting from 7 days after nerve ligation. We did not use MSOP in these experiments because the drug is not systemically active. We assessed mechanical pain threshold before ligation of the sciatic nerve, 1 day prior to the onset of drug treatments, and 1 h after the last administration (day 14 after nerve ligation). As opposed to what observed after a single injection, repeated CA injections did not cause analgesia in CCI mice (0.127 ± 0.062 g, *p* = 0.89) as compared to CCI mice treated with vehicle (0.08 ± 0.021 g) (Figure 6), suggesting the development of tolerance. However, the analgesic activity of CA was disclosed when the drug was combined with systemic CH223191 (0.419 ± 0.11 g), which was inactive on its own (0.058 ± 0.015 g) (Figure 6).

#### Electrophysiological Activity of Spinal Nociceptive Specific Neurons in Wild-Type Chronic Constriction Injury Mice

**FIGURE 7:**
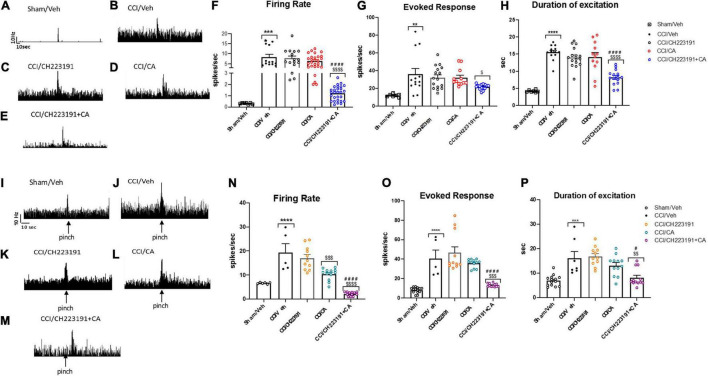
Spinal NS neurons and RVM ON cell hyperexcitability is significantly attenuated by repeated administration of CA in combination with CH223191, in WT CCI mice. **(A–E)** Representative ratemeters showing spontaneous and noxious-evoked activity of spinal NS neurons in Sham mice **(A)** or in CCI mice before and after treatment with vehicle **(B)**, CH223191 **(C)**, CA (0.25 mg/Kg) **(D)**, CH223191+CA (E). **(F–H)** Mean ± S.E.M population data of systemic repeated administrations of CH223191 (1 mg/Kg) in presence of cinnabarinic acid (CA, 0.25 mg/Kg) on firing rate **(F)**, evoked response **(G)** and duration of excitation **(H)** of spinal NS neurons, in CCI mice **(I–M)** representative ratemeters showing spontaneous and noxious-evoked activity of RVM ON cell in Sham mice **(I)** or in CCI mice before and after treatment with vehicle **(J)**, CH223191 **(K)**, CA (0.25 mg/Kg) **(L)**, CH223191+CA **(M)**. **(N–P)** Mean ± S.E.M population data of systemic repeated administrations of CH223191 (1 mg/Kg) in presence of cinnabarinic acid (CA, 0.25 mg/Kg) on firing rate **(N)**, evoked response **(O)** and duration of excitation **(P)** of RVM ON cell, in CCI mice. Each point represents the mean of five different mice per group (one neuron recorded per each mouse). **p < 0.01, ***p < 0.001 and ****p < 0.0001 indicate statistically significant difference vs. Sham/Veh, ^$^*p* < 0.05, ^$$^*p* < 0.01, ^$$$^*p* < 0.001 and ^$$$$^*p* < 0.0001 indicate statistically significant difference vs. CCI/CH223191, ^#^*p* < 0.05 and ^####^*p* < 0.0001 indicate statistically significant difference vs. CCI/CA. One-way ANOVA followed by Tukey’s *post-hoc* test for multiple comparisons was used.

Repeated injections of CA or CH223191 alone did not affect the activity of spinal NS neurons in CCI mice. However, the combination of the two drugs was able to significantly attenuate CCI-induced spinal hyperexcitability. NS neurons of CCI mice treated with vehicle showed a spontaneous activity of 8.34 ± 1.39 spikes/s, a frequency of excitation of 35.92 ± 6.39 spikes/s and the duration of excitation of 15.52 ± 0.6 s (Figures 7F–H). In response to a combined treatment with CA and CH223191, NS neurons showed a spontaneous activity of 1.21 ± 0.63 spikes/s (*p* < 0.0001, *n* = 5), a frequency of excitation of 21.72 ± 0.76 spikes/s (*p* = 0.09, *n* = 5), and a duration of excitation of 8.18 ± 0.75 s (*p* < 0.0001, *n* = 5) (Figures 7F–H). NS neurons of mice treated with CA alone showed a spontaneous activity of 6.41 ± 0.6 spikes/s (*p* = 0.36, *n* = 5), a frequency of excitation of 32 ± 2.85 spikes/s (*p* = 0.94, *n* = 5) and the duration of excitation of 14.15 ± 1.33 s (*p* = 0.76, *n* = 5) (Figures 7F–H), whereas, NS neurons of CCI mice treated with CH223191 showed a spontaneous activity of 7.73 ± 1 spikes/s (*p* = 0.98, *n* = 5), a frequency of excitation of 32.26 ± 3.15 spikes/s (*p* = 0.94, *n* = 5), and a duration of excitation of 14.05 ± 0.71 s (*p* = 0.65, *n* = 5) (Figures 7F–H). Population data of NS neurons in Sham mice or in CCI mice before and after treatment with vehicle, CA (0.25 mg/Kg), CH 223191, CH223191+CA are represented in Figures 7F,G.

#### Electrophysiological Activity of Rostral Ventromedial Medulla ON Cells in Wild-Type Chronic Constriction Injury Mice

As opposed to data obtained in the spinal cord, repeated injections with CA significantly reduced the firing rate of ON cells (10.37 ± 0.71 spikes/s, *p* = 0.0005, *n* = 5) in the RVM of CCI mice (Figures 7L–N), as compared to vehicle-injected CCI mice (19.38 ± 3.66 spikes/s, *n* = 5) (Figures 7I,J). No differences were observed neither in the frequency nor in the duration of excitation between CCI mice treated with vehicle (40.67 ± 8.49 spikes/s for frequency and 16.08 ± 2.80 s for duration of excitation, respectively) or with CA (35.66 ± 1.24 spikes/s for frequency, *p* = 0.90 and 13.11 ± 1.19 s, for duration of excitation, *p* = 0.56) (Figures 7K–M). Repeated injections of CH223191 alone did not change any of these parameters (17.03 ± 1.47 spikes/s, 46.66 ± 6 spikes/s, and 16.86 ± 1.18 s) (Figures 7K–M). In contrast, the combined treatment with CA and CH223191 markedly reduced the firing rate (2.15 ± 0.23 spikes/s, *p* < 0.0001) and the frequency (13.16 ± 0.49 spikes/s, *p* < 0.0001) and duration (19.86 ± 1.18 s, *p* = 0.038) of excitation of RVM ON cells in CCI mice (Figures 7K–M). In Sham mice treated with vehicle, ON cell showed a spontaneous activity of 19.38 ± 3.66 spikes/s (*p* = 0.0005, *n* = 5), a frequency of excitation of 40.67 ± 8.49 spikes/s (*p* = 0.90, *n* = 5) and a duration of excitation of 16.08 ± 2.80 spikes/s (*p* < 0.0001, *n* = 5). Population data of ON cell in Sham mice or in CCI mice before and after treatment with vehicle, CA (0.25 mg/Kg), CH 223191, CH223191+CA are represented in Figures 7N–P.

Taken together, these findings demonstrate that (i) repeated CA injections fail to cause analgesia in the CCI model of neuropathic pain unless combined with pharmacological blockade of Ah receptors; and (ii) this combination causes a large reduction in the activity of nociceptive spinal cord neurons and ON RVM cells.

## Discussion

We moved from the evidence that the mGlu4 receptor, one of the putative CA receptor target (Fazio et al., 2012), has been found to be involved in the pathophysiology of tonic and chronic pain in several regions of the pain neuraxis (Vilar et al., 2013; Zussy et al., 2018). Here, we have shown for the first time that CA causes analgesia in the formalin model of inflammatory pain, and in the CCI model of neuropathic pain. Different doses of CA were tested in the formalin test, and we were surprised to find that the compound caused analgesia at doses of 0.125 and 0.25 mg/kg, but not at higher doses. This is unusual for a kynurenine metabolite, because most of the kynurenines are behaviourally active at doses >10 mg/kg (Lapin, 1998; Fazio et al., 2015; Varga et al., 2015). CA displayed also a great potency in behavioural models predictive of antipsychotic-like activity in which, however, the compound retained its action at doses of 0.5, 1, and 5 mg/kg (Ulivieri et al., 2020). It is possible that doses of CA > 0.25 mg/kg cause a rapid desensitization of the receptor(s) responsible for CA-induced analgesia, or, alternatively, recruit additional targets that counterbalance the analgesic activity. CA can also activate the aryl hydrocarbon receptor (Ah receptor), an intracellular receptor, which is known to translocate into the nucleus and regulate gene expression in response to agonist activation (Joshi et al., 2017). For example, CA can enhance IL-22 production in human T cells by activating Ah receptor (Godefroy et al., 1990). CA-induced analgesia in the formalin test was abrogated in mGlu4 knockout mice, but was unaffected by treatment with the Ah receptor antagonist, CH223191. Thus, it appear that CA-induced analgesia in the second phase of the formalin test was mediated by activation of mGlu4 receptors, but did not involve the Ah receptor. It is intriguing that CA shows a lower potency than glutamate in activating mGlu4 receptors (Fazio et al., 2012), and that low doses of CA caused analgesia in spite of the competition with synaptically released glutamate. One possibility is that CA is not cleared from the synapse as efficiently as glutamate, and activation of mGlu4 receptors by CA is long-lasting. This might reinforce the ability of presynaptic mGlu4 receptors to restrain the release of glutamate and other neurotransmitters/neuropeptides involved in pain transmission.

We examined in more detail the action of CA in the CCI model of neuropathic pain, in which nociceptive sensitization reflects maladaptive changes in synaptic plasticity different stations of the pain neuraxis associated with neuroinflammation mediated by astrocytes and microglia in the spinal cord (Li et al., 2016; Dubovı et al., 2018; Shu et al., 2020). We could confirm the analgesic activity of acute CA injection CCI model. To further strengthen the behavioural data, we also measured the ongoing and evoked activity of (i) nociceptive specific neurons (NS) in the spinal cord, and (ii) RVM ON following PAG stimulation. Interestingly, CA injection did not change the activity of nociceptive neurons in the spinal cord, but reduced the duration of excitation of RVM ON cells. Thus, it is likely that CA acted in the RVM and perhaps in other supraspinal regions to enhance mechanical thresholds in the CCI model. The RVM is a key region in the top-down regulation of pain, and inhibition of RVM ON cells fits nicely with the analgesic activity of CA. Interestingly, however, an action of CA in the spinal cord was disclosed by local application of MSOP or CH223191, which were inactive on their own, but respectively enhanced and reduced the activity of spinal neurons when combined with systemic CA. MSOP is a non-selective antagonist of mGlu4, - 6-, -7, and -8 receptors (Nicoletti et al., 2011), however the interaction with CA likely involves the mGlu4 receptor because CA has no activity at mGlu6, -7 or -8 receptors (Fazio et al., 2012). The effects of MSOP and CH223191 in the spinal cord suggest that the two receptors (mGlu4 and Ah receptor) recruited by CA have opposite function in pain control, and a suppressive activity of CA on nociceptive neurons can only be unmasked if the Ah receptor is pharmacologically inhibited. A similar scenario was seen in the PAG/RVM, where CA largely reduced all parameters of ON cell activity when Ah receptor was blocked by application of CH223191 in the PAG.

As opposed to the acute treatment, repeated injections of CA did not affect pain thresholds in the CCI model, suggesting the development of tolerance. Mechanisms of pharmacokinetic tolerance, pharmacodynamics tolerance, or pseudo-tolerance may account for the loss of analgesia in response to repeated injections of CA. In mice injected i.p. with 0.25 mg/kg CA, serum CA levels peak after 15 min, are largely reduced at 60 min, and returned back to normal values at 120 min, whereas levels in the cerebral cortex and cerebellum remain high for at least 12 h (Ulivieri et al., 2020). An accurate PK analysis is needed to establish whether CA exposure decreases in response to repeated injections. Interestingly, CA-induced analgesia was unmasked when the AhR antagonist, CH223191, was co-injected with CA for 7 days. This was paralleled by the activity of nociceptive spinal neurons and RVM ON cells, which was largely reduced in CCI mice treated with CA *plus* CH223191, although CA alone significantly reduced the firing rate of RVM ON cells. A possible explanation is that the Ah receptor is up-regulated in the pain neuraxis in response to CA administration, or, alternatively, that the “analgesic” mGlu4 receptor is down-regulated. This hypotheses warrant further investigation. Finally, we cannot exclude that CA accumulating in brain tissue as a result of repeated injections reaches the threshold to recruit additional targets that counteract CA-induced analgesia. The existence of these targets has been hypothesized (Fazio et al., 2012), but their identity is unknown.

Taken together these data show for the first time that the trace kynurenine, CA, causes analgesia through a mechanism mediated, at least in part, by the activation of mGlu4 receptors. The high potency of CA in reducing nocifensive behaviour in the formalin test or enhancing mechanical thresholds in the CCI model suggests that the small amounts of CA endogenously produced by the kynurenine pathway of tryptophan metabolism may act physiologically to regulate pain transmission. The translational value of our findings for pain treatment in humans is limited because of the lack of analgesia with increasing doses of CA and the development of tolerance in response to repeated administrations. However, it will be interesting to examine whether changes in endogenous CA levels, which are detectable in the peripheral blood, are associated with inter-individual variability in pain sensitivity. Because activation of the kynurenine pathway is one of the putative communication systems in the gut-brain axis (Dehhaghi et al., 2019; Roth et al., 2021), the evidence that CA modulates pain transmission may provide a novel link between the gut microbiota and inflammatory or neuropathic pain.

## Perspectives

Studying the role of the endogenous metabolite cinnabarinic acid, in the pain pathways as a new neurotransmitter of the central nervous system can lay the foundations for new therapies in the treatment of painful pathologies, as well as increase the available knowledge about new pain-related endogenous processes.

## Data Availability Statement

The raw data supporting the conclusions of this article will be made available by the authors, without undue reservation.

## Ethics Statement

The animal study was reviewed and approved by Italian Ministry of Health 5015/2021-PR.

## Author Contributions

FN, SM, LL, GB, and VB: conceptualization and writing. SN, SB, NA, FR, GM, FF, MS, PS, and FL: experiments, data analysis, and writing. All authors contributed to the article and approved the submitted version.

## Conflict of Interest

The authors declare that the research was conducted in the absence of any commercial or financial relationships that could be construed as a potential conflict of interest.

## Publisher’s Note

All claims expressed in this article are solely those of the authors and do not necessarily represent those of their affiliated organizations, or those of the publisher, the editors and the reviewers. Any product that may be evaluated in this article, or claim that may be made by its manufacturer, is not guaranteed or endorsed by the publisher.
